# PG545, a Heparan Sulfate Mimetic, Reduces Heparanase Expression *In Vivo*, Blocks Spontaneous Metastases and Enhances Overall Survival in the 4T1 Breast Carcinoma Model

**DOI:** 10.1371/journal.pone.0052175

**Published:** 2012-12-26

**Authors:** Edward Hammond, Ralf Brandt, Keith Dredge

**Affiliations:** 1 Research and Development, Progen Pharmaceuticals, Darra, Queensland, Australia; 2 vivoPharm, Bundoora, Victoria, Australia; University of Patras, Greece

## Abstract

PG545 is a clinically relevant heparan sulfate (HS) mimetic which, in addition to possessing anti-angiogenic properties, also acts as a heparanase inhibitor which may differentiate its mechanism(s) of action from approved angiogenesis inhibitors. The degradation of HS by heparanase has been strongly implicated in cell dissemination and the metastatic process. Thus, the anti-metastatic activity of PG545 has been linked to the enzymatic function of heparanase – the only endoglycosidase known to cleave HS, an important component of the extracellular matrix (ECM) which represents a potential avenue for therapeutic intervention for certain metastatic cancer indications. Recent concerns raised about the paucity of overall survival as an endpoint in mouse models of clinically relevant metastasis led us to examine the effect of PG545 on the progression of both primary tumor growth and the spontaneously metastasizing disease in the 4T1 syngeneic breast carcinoma model in a non-surgical and surgical (mastectomy) setting. PG545 significantly inhibited primary tumor growth but importantly also inhibited lung metastasis in treated mice, an effect not observed with the tyrosine kinase inhibitor sorafenib. Importantly, PG545 significantly enhanced overall survival compared to vehicle control and the sorafenib group, suggesting PG545’s inhibitory effect on heparanase is indeed a critical attribute to induce anti-metastatic activity. In addition to blocking a common angiogenic signalling pathway in tumor cells, the expression of heparanase in the primary tumor and lung was also significantly reduced by PG545 treatment. These results support the ongoing development of PG545 and highlight the potential utility in metastatic disease settings.

## Introduction

Metastasis is the leading cause of cancer death [1]. According to the American Cancer Society, breast cancer is expected to account for 30% (230,480) of all new cancer cases among women [2]. Approximately 6–10% of patients have metastatic disease at the time of diagnosis and 30% who are initially diagnosed with earlier-stage breast cancer will eventually develop recurrent advanced or metastatic disease [3]. The prognosis for these patients is poor, with an estimated 5-year survival of only 21% [4]. The primary goal of treatment must be palliation of disease where feasible, but this should not be pursued at the expense of unmanageable toxicity [5]. In addition to toxicity issues, the standard developmental approach to the treatment of micrometastatic disease assumes a progression from Phase I to Phase III trials in the metastatic setting, followed by a transition to large proof-of-concept adjuvant trials. This approach may miss important opportunities by not focusing on the metastatic cascade and not all agents successful in the overt metastatic setting are useful in the micrometastatic disease setting; similarly, not all agents successful in the micrometastatic setting would be beneficial in the macrometastatic setting [1]. Among 37 phase III trials conducted in the last 15 years, only three systemic therapies were approved for first-line use and nine were approved for use as second-line or other lines of therapy. Of these, only four were supported by results showing longer survival times [6], illustrating a clear need to better assess the anti-metastatic effect of cancer therapeutics and improve overall survival rates.

Heparanase is an endo-β-glucuronidase that degrades heparan sulfate (HS), a major constituent of the extracellular matrix (ECM) and basement membrane, and this enzyme plays a role in tumor metastasis and angiogenesis [7]–[9]. The cleavage of HS chains by heparanase not only facilitates migration of tumor cells through the disruption of the ECM but also results in the release of signalling proteins (typically stored bound to HS) which can then bind to their corresponding receptors to initiate signal transduction, thereby promoting cancer growth, angiogenesis and invasion [10], [11]. Heparanase is also thought to have a role in proliferative signalling that is distinct from its HS-degrading activity [12], [13]. This protein has been widely implicated as an important regulator of proliferation, invasion, metastasis and malignancy-associated angiogenesis in several tumor types including breast cancer and its presence is a key indicator of malignancy in this disease [9], [10], [14]–[17]. In a clinical study, heparanase expression was significantly upregulated in microinvasive lesions in ductal carcinoma *in situ* [18]. A tumor-inducing effect on heparanase expression by lymphocytes of breast cancer patients was found to be decreased in breast cancer patients rendered free of tumor by surgery or treated with tamoxifen [19]. Given the multitude of functions of heparanase, systemic heparanase amplification can perpetuate tumor-promoting autocrine, paracrine, and growth factor signalling, making heparanase inhibitors potentially effective against invasive cancers [20].

A number of cancer therapeutics under development have been designed to target heparanase [8] and recent identification of micro RNA mechanisms linked to brain metastatic breast cancer through heparanase control offers further rationale to develop heparanase-based therapeutics [21]. Although not specifically under development for breast cancer, heparanase inhibitors have entered Phase II clinical trials, with the sulfated oligosaccharide PI-88 demonstrating activity in hepatocellular carcinoma [22]. Earlier stage compounds such as sulfated hexasaccharides have recently been shown to inhibit heparanase and attenuate metastasis in B16-BL6 melanoma cells (high heparanase expressing cells) but no effect on the metastasis of MC-38 carcinoma cells (which express little or no heparanase) [23]. SST0001 is an N-acetylated, glycol-split high molecular weight heparin that also exhibits low anticoagulant activity, selectively inhibits heparanase and has shown activity in an *in vivo* model of multiple myeloma [24]. M402, a HS mimetic designed to inhibit multiple factors implicated in tumor-host cell interactions, including heparanase, showed some survival benefit in an orthotopic 4T1 murine mammary carcinoma model [25]. Taken together, the inhibition of heparanase by these HS mimetics may be the key differentiating factor – in addition to their ability to inhibit vascular endothelial growth factor (VEGF) by targeting its HS-binding – to explain their anti-metastatic properties in contrast to some VEGF inhibitors and tyrosine kinase inhibitors (TKIs) which arguably lead to divergent effects on metastasis [26].

Here we report the antitumor and antimetastatic properties of PG545, a synthetic, fully sulfated HS mimetic, in a 4T1 breast carcinoma model to demonstrate the potential utility of this approach for patients who undergo surgery of the primary tumors or those with a high risk of recurrence or limited metastatic disease. PG545 is known to inhibit the enzymatic activity of heparanase, the signalling of angiogenic growth factors, *in vitro* angiogenesis, solid tumor growth and blockade of lung colonisation in an experimental metastasis model [27]. Subsequent studies confirmed anti-angiogenic activity *in vivo* and, in addition to significant effects on solid tumor progression, potent anti-metastatic activity was confirmed in spontaneously arising metastatic models of colon and lung cancer [28]. While this antimetastatic activity is likely mediated by the inhibition of heparanase activity, PG545 also inhibits growth factors such as VEGF and fibroblast growth factor 2 (FGF-2) that promote heparanase expression [29], [30]. So, PG545 may suppress metastasis by direct inhibition of heparanase activity or by reducing its expression. To date, there are very few preclinical studies that have been conducted with antiangiogenic drugs that analyse neoadjuvant or pre-surgical treatments and virtually none that compare treatment effects on primary tumors to metastatic-disease progression (or prevention) after surgery [31]. Thus, to study the effect of PG545 on metastasis and overall survival following mastectomy, we employed the syngeneic orthotopic 4T1 breast cancer model because 4T1 cells are aggressively metastatic, forming characteristic large lung metastases rapidly which lead to 90% mortality within 70 days [32]. Moreover, the 4T1 model is known to be one of the few breast cancer models with the capacity to metastasize efficiently to sites affected in human breast cancer [33]. Given that PG545 has anti-angiogenic properties and that there are recent data showing divergent metastatic effects with other angiogenesis inhibitors [28], [34], [35], we also included an angiogenesis inhibitor, sorafenib, in the model to assess its effect on metastasis. Finally, we also investigated the mechanism of action relating to the antimetastatic and antiangiogenic effect of PG545 by assessing the *in vivo* levels of heparanase, phosphorylated extracellular signal-regulated kinase (ERK)1/2, VEGF and FGF-2.

## Materials and Methods

### Drugs and Reagents

PG545 is a fully sulfated tetrasaccharide functionalized with a cholestanyl aglycon designed at Progen Pharmaceuticals Ltd (Brisbane, QLD, Australia). Sorafenib was sourced from Bayer Health Care (Leverkusen, Germany) and cisplatin clinical formulation (1.0 mg/mL) from Pfizer (West Ryde, NSW, Australia). PG545 was dissolved using cell culture medium for *in vitro* experiments and phosphate buffered saline (PBS) pH 7.2 for *in vivo* studies to a final concentration of 2.5 or 2.0 mg/mL (or 1.0 mg/mL upon reduction of dose in the first survival study). The dosing solutions were prepared fresh on each day of dosing. Sorafenib was supplied as 315 mg tablets containing 200 mg active sorafenib. Tablets were crushed and dissolved in N-Methyl-2-pyrrolidone (NMP) to formulate stock solution of active sorafenib. This stock solution was further diluted with polyethylene glycol (PEG) 300 to achieve the required dose concentration. NMP, PEG 300 and PBS were obtained from Sigma-Aldrich (Castle Hill, NSW, Australia).

### Cell Lines

4T1 mouse breast cancer cells were sourced from American Type Culture Collection (ATCC) (Rockville, MD, USA). Reagents for culture of 4T1 mouse breast cancer cells were obtained from the following suppliers: RPMI 1640 cell culture medium, fetal bovine serum (FBS) and Hank’s balanced salt solution (HBSS) from Invitrogen Australia (Mt Waverley, VIC, Australia); penicillin-streptomycin and Trypan Blue from Sigma-Aldrich (Castle Hill, NSW, Australia). 4T1 mouse breast cancer cells (Passage 3 from working stock VP-Stock 314) were cultured in RPMI 1640 cell culture medium, supplemented with 10% FBS and 50 IU/mL penicillin-streptomycin. The cells were harvested by trypsinization, washed twice in HBSS and counted. The cells were then resuspended in HBSS to a final concentration of 5 × 10^7^ cells/mL. Cells were cultured at 37°C in a humidified cell culture incubator supplied with 95% air/5% CO_2_.

### *In vivo* Models

Procedures involving the care and use of animals in this study were reviewed and approved by the University of Adelaide (South Australia) Animal Ethics Committee prior to conduct (Certificate Number: M46-2008). During the study, the care and use of animals was conducted in accordance with the principles outlined in the Australian Code of Practice for the Care and Use of Animals for Scientific Purposes, 7th Edition, 2004 (National Health and Medical Research Council). All surgery was performed using Ketamil (10 mg/mL)/Xylazil (0.9 mg/mL) as the anaesthetic and all efforts were made to minimize suffering. Female BALB/c mice (Animal Resource Centre, Canning Vale, WA, Australia) were used for all the *in vivo* experiments. For the primary tumor/metastases model, 10 µL of 4T1 cells (5×10^5^ cells) were discharged directly into the third mammary fat pad and treatment of mice began 7 days after 4T1 cell inoculation, when the average tumor volume was approximately 100 mm^3^. For the survival experiments, a refined 4T1 inoculation method used Matrigel (BD Biosciences, North Ryde, NSW, Australia) and 10 µL of 4T1 cells (1×10^5^ cells) introduced directly into the fourth mammary fat pad. Mice were randomized, based on tumor size, into two groups of twenty mice and four groups of fifteen mice, five days post-inoculation (Day 0). On Day 6 of the study (eleven days post-inoculation) mastectomy was performed on all mice while under injectable Ketamine (10 mg/mL)/Xylazine (0.9 mg/mL) anaesthetic. The fourth mammary fat pad including the primary tumor was excised. The dosing regimen used in each experiment is outlined in the figures. We previously demonstrated that PG545 administered up to 25 mg/kg twice weekly potently blocks angiogenesis *in vivo* and 20 mg/kg once weekly effectively suppresses both tumor growth and metastasis [28]. In the non-mastectomy study, primary tumors were measured in three dimensions on a twice weekly schedule (length and width) and the tumour volume (cm^3^) calculated using the equation V = length × width^2^ × π/6 and the anti-tumour activity was expressed as percent tumour growth inhibition (%TGI) using an equation previously described [28] while tumors in survival studies were excised at mastectomy from mice in the vehicle control and PG545 monotherapy groups and weighed. The lungs were excised from all mice at the time of culling. The number of overt macrometastases of the surface of the lungs were enumerated manually [36] from mice in the vehicle control and PG545 monotherapy groups, and expressed as inhibition rate (IR) as previously described [37] or, where distinct, were categorized according to size: small (<1 mm), medium (≥1 mm and <3 mm) and large (≥3 mm) as previously described elsewhere [32].

During the main mastectomy study, animals were monitored closely and euthanized when displaying signs of distress in accordance with local and other guidelines [38] or until the study was terminated on day 55. Based on previous pilot studies and coinciding with the time point when eight mice from the vehicle control group were removed from the study due to disease progression (Day 30), five mice each were randomly selected from each group and culled for collection of plasma (from terminal cardiac blood sample) and excision of lungs. These mice, referred to as satellite mice, were not included in the survival part of the study. The same samples were also collected from five non-inoculated, aged-matched, untreated mice for baseline comparison. Correlation between lung metastasis counts and lung weight was determined as an additional parameter as a measure of metastatic burden.

### Histopathology and Immunohistochemistry

Formalin-fixed tumour tissue and lungs from each mouse in the vehicle control and PG545 monotherapy groups were paraffin embedded. Sections were stained with haematoxylin and eosin (H&E) for microscopic enumeration of lung micrometastases. Formalin fixed lungs from all other mice were stored at ambient temperature for potential future analysis. The rabbit polyclonal heparanase antibody (Abcam, Cambridge, MA, USA) was used in Immunohistochemical (IHC) analysis of mouse tumour and lung sections. Paraffin-embedded mouse tissue sections were deparaffinized and endogenous peroxidase activity was quenched by incubating the sections in 0.5% hydrogen peroxide in methanol for 30 minutes. Following two washes in PBS, antigen retrieval was performed by boiling the sections in 0.037% EDTA pH 8.0 for 10 minutes. Sections were then washed in PBS twice and then blocked in 3% normal horse serum for 30 minutes followed by an overnight incubation at a dilution of 1/500 heparanase antibody in 3% normal horse serum. The following day, slides were washed twice in PBS followed by development with the UltraVision HRP Polymer detection system and DAB Plus substrate (Thermo Scientific, Scoresby, VIC, Australia). Sections were then washed in running tap water then counterstained in Mayer’s hematoxylin solution, dehydrated, cleared, and mounted in Depex mounting medium (VWR, West Chester, PA, USA) The sections were viewed under an Olympus BX51 microscope using a 40× objective lens and images were captured using an electronic eyepiece and an Olympus DP20 camera system.

### Enzyme-linked Immunosorbent Assay (ELISA)

The levels of heparanase, VEGF and FGF-2 in excised primary tumors were quantified by subjecting tumor tissue homogenates to ELISA. Tumor tissue was homogenized on ice and extracted into ice cold buffer consisting of, 50 mM Tris HCl pH 7.4, 150 mM NaCl, 1% Triton X-100, 1% Halt protease and phosphatase inhibitor cocktail (Thermo Scientific, Scoresby, VIC, Australia). The homogenates were extracted at 4°C for 90 min before being clarified by centrifugation at 13,000 g for 20 min at 4°C. Protein concentration in the homogenates was determined using a Coomassie dye assay (Thermo Scientific, Scoresby, VIC, Australia) and they were stored at −80°C until further analysis. The concentration of heparanase, VEGF, FGF-2 and the abundance of the phosphorylated forms of ERK1 (T202 or Y204) and ERK2 (T105 or Y187) in the tumor tissue homogenates was measured using ELISA kits specific for the mouse versions of these proteins: heparanase, USCN, Wuhan, China; VEGF, R&D Systems, Minneapolis, MN, USA; FGF-2 and p-ERK1/2 (Abcam, Cambridge, MA, USA). In each case, ELISAs were performed according to the manufacturer’s instructions.

### Statistics

A one-way analysis of variance (ANOVA) was performed on tumor volumes or surface lung metastases at the end of the first study followed by either a Holm-Sidak method or Dunnett’s Method were performed. In the mastectomy study, a t-test was used to determine the significance of the difference in tumour weight at mastectomy, lung weights, surface and micrometastases from satellite mice between control and the PG545 group. Survival data were first analysed by Kaplan‐Meier analysis using log ranks of survival. The differences between levels of heparanase, VEGF, FGF-2 and p-ERK 1/2 in control and PG545 groups were analysed using t-tests. P<0.05 was considered significant in all analyses. All statistical calculations were performed using either SigmaPlot or GraphPad Prism 5.0.

## Results

### PG545 Inhibits Primary Tumor Growth and Spontaneous Metastasis in the Orthotopic 4T1 Model

**Figure 1:**
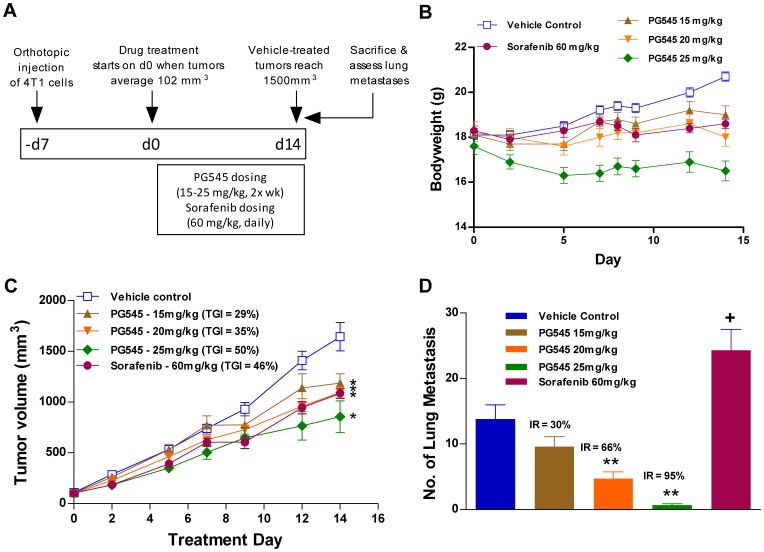
PG545 inhibits primary tumor growth and spontaneous metastasis to lung in 4T1 breast cancer model. (**A**) Experimental protocol for the orthotopic 4T1 primary tumor and spontaneous metastasis model. Female Balb/c mice were injected with 10 µL of 5×10^5^ 4T1 cells into the third mammary fat pad. Twice-weekly treatment with PG545 commenced once the primary tumors reached a size of approximately 100 mm^3^ (n = 10). (**B**) Twice weekly treatment with PG545 at 25 mg/kg led to a non-significant bodyweight loss of 4.7% by day 14. (**C**) PG545 significantly inhibited the growth of the primary 4T1 tumor. The study ended on treatment day 14 due to serious clinical signs in some groups. (**D**) On treatment day 14, mice were examined for the presence of metastasis in the lungs and a dose-dependent inhibitory effect with PG545 was apparent while sorafenib significantly increased the number of lung metastases. A one-way ANOVA was performed on tumor volumes or surface lung metastases counts measured in all surviving mice at the end of the study followed by either a Holm-Sidak method or Dunn’s Method were performed. * = P<0.05, ** = P<0.01 for PG545-treated groups versus vehicle control and + = P<0.05 for sorafenib-treated group versus vehicle control.

The experimental protocol for the spontaneous orthotopic 4T1 model is presented in Figure 1A. A twice-weekly dosing regimen with PG545 resulted in an average bodyweight loss up to 4.7% by the end of the study on day 14 (Figure 1B). PG545 treatment led to a significant inhibition of the primary tumor growth at all doses tested, producing TGI values of 29, 35 and 50% for 15, 20 and 25 mg/kg respectively (Figure 1C). Daily dosing with sorafenib (60 mg/kg) also significantly inhibited tumor progression, producing a TGI value of 46%. At the end of the study on day 14, mice were examined for evidence of spontaneous metastasis to the lung. PG545 significantly suppressed the formation of surface lung metastases in a dose-dependent manner, producing IR values of 30, 66 and 95% for 15, 20 and 25 mg/kg PG545 respectively (Figure 1D). In contrast, sorafenib significantly increased the number of lung metastases compared to vehicle control.

### PG545 Significantly Prolongs Overall Survival in the 4T1 Mastectomy Model

**Figure 2:**
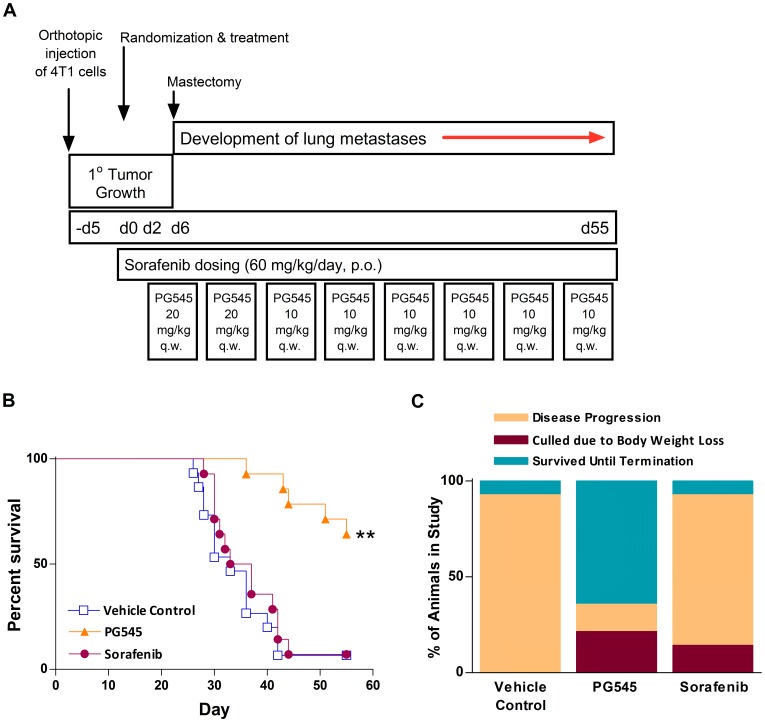
PG545 significantly enhances overall survival in a mastectomy model of 4T1 breast cancer model. (**A**) Experimental protocol for the mastectomy model. Female Balb/c mice were injected with 10 µL of 1×10^5^ 4T1 cells into the fourth mammary fat pad and were randomised (n = 20 per group), based on tumour size, five days post-inoculation (Day 0). On Day 6 of the study mastectomy and the dosing regimen is shown. (**B**) The number of mice surviving at the end of the study was significantly greater in the PG545-treated group compared to vehicle control. Sorafenib appeared no more effective than the vehicle control group. (**C**) The reasons for death during the study are illustrated as percentage of animals in the study who were euthanized due to disease progression, culled due to excessive bodyweight loss or survived until termination. The once-weekly dosing regimen with PG545 resulted in an average bodyweight loss of 12%, once daily treatment with sorafenib led to an average bodyweight loss of 14% by day 55. Survival data were first analysed by Kaplan‐Meier analysis using Log ranks of survival. ** = P<0.01 versus vehicle control.

PG545 and sorafenib were administered as outlined in the experimental protocol shown in Figure 2A. PG545 significantly enhanced the overall survival of mice compared with vehicle control and was comparable to cisplatin (Figure S1) which has been published as an active cytotoxic agent by other groups in this model [25], [32], but sorafenib had no effect on overall survival (Figure 2B). The once-weekly dosing regimen with PG545 resulted in an average bodyweight loss of 12% by the end of the study on day 55 compared to a loss of 14% with daily sorafenib treatment. Although the effects of these agents on bodyweight is significant, it is likely enhanced by the requirement for surgery and the duration of the model. Moreover, PG545 effects on bodyweight may be species or strain-dependent and influenced by tumor-bearing animals. At comparable and higher human equivalent doses (HED) in definitive rat and dog toxicology studies, PG545-induced reductions in bodyweight were not apparent with the main toxicities associated with elevation of serum lipids, vacuolation in tissues, increases in leucocyte counts, decreases in erythrocytes and platelets (Table S1). For the longer term mouse survival studies, a pilot study identified that a loading dose of 20 mg/kg followed weekly maintenance doses of 10 mg/kg was sufficient to ensure efficacious plasma concentrations of PG545 (Figure S2). By the end of the study on day 55, only 1/14 vehicle control-treated mice (7%) remained on study. Similarly, only 1/14 mice (7%) in the sorafenib group survived until the end of the study although 14% were euthanized due to bodyweight loss as opposed to disease progression. In contrast, treatment with PG545 ensured survival for 9/14 or 64% of the animals (Figure 2C).

Table 1 shows that although 64% of PG545-treated mice survived until the end of the study, 21% were culled due to bodyweight loss while a further 14% were culled due to disease progression – however that is in comparison to the 93% of mice in the vehicle control group and 79% in the sorafenib monotherapy group which were also culled due to clinical signs of disease progression. Thus, only 7% of sorafenib-treated mice survived until the end of study.

**Table 1:** Summary of the performance of PG545 and sorafenib in the 4T1 mastectomy model.

Treatment	% Mice Culled Due to Excess (>15%) Body Weight Loss	% Disease Progression	% Survivors
Vehicle Control	0 (0)	93 (13)	7 (1)
PG545	21 (3)	14 (2)	64 (9)
Sorafenib	14 (2)	79 (11)	7 (1)

Data are percentages with absolute numbers of mice in parentheses.

### PG545 Significantly Inhibits Metastasis to the Lung but also Reduces Primary Tumor Growth in the 4T1 Mastectomy Model

**Figure 3:**
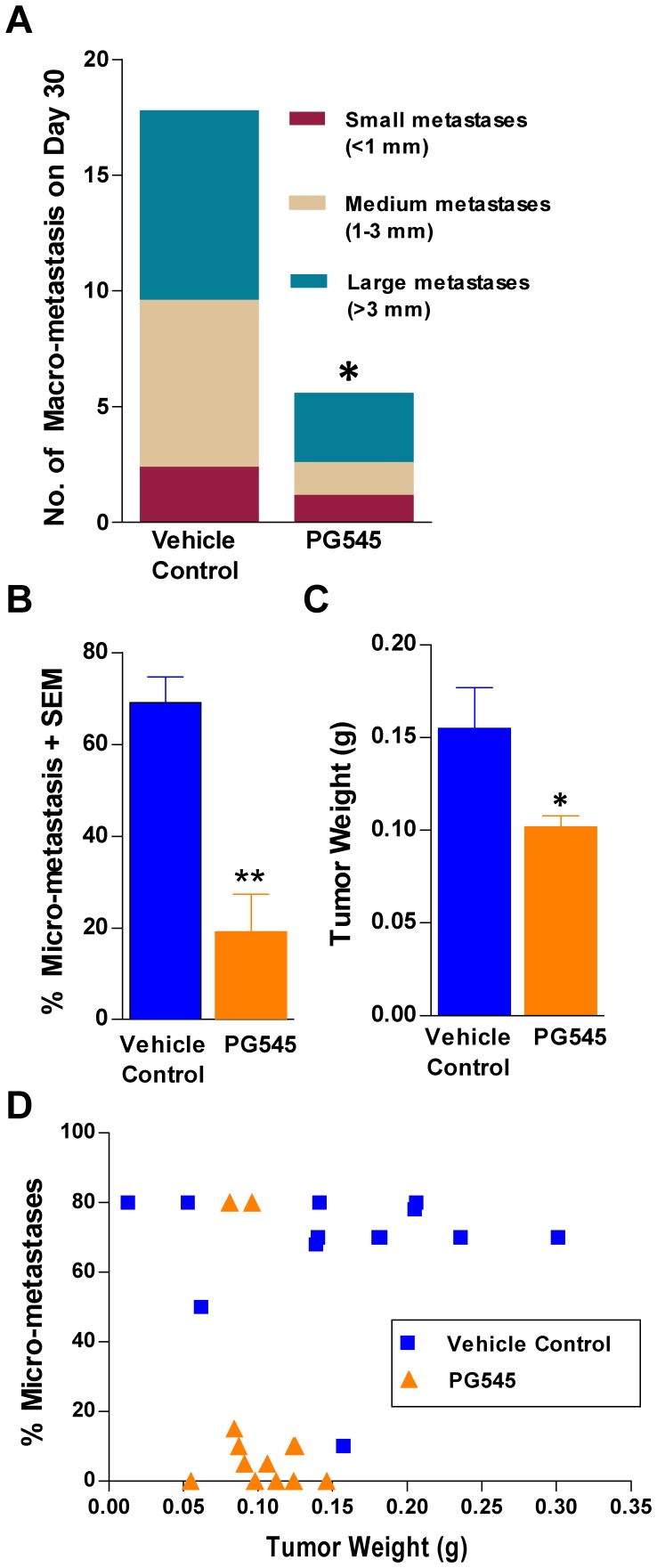
PG545 significantly inhibits lung macrometastases, lung micrometastases and primary tumor growth in 4T1 mastectomy model. (**A**) In satellite mice (n = 5), the number of large surface lung metastases were significantly reduced following treatment with PG545 compared with vehicle controls. (**B**) PG545 also significantly inhibited the percentage of micrometastases as identified by histological assessment. (**C**) PG545 significantly inhibited the size of the primary tumor as weighed following the mastectomy procedure. (**D**) Lung micrometastases plotted against primary tumor size in vehicle control (open squares) and PG545 (closed triangles) revealed that despite PG545 significantly inhibiting tumor weight at mastectomy, no correlation was found between the size of the primary tumor and lung micrometastases in individual mice in control or PG545-treated groups (R^2^ = 0.1). A t-test was used to determine the significance of the difference in tumour weight at mastectomy, surface lung metastases and micrometastases between control and the PG545 group. * = P<0.05, ** = P<0.01 versus vehicle control.

On day 30, a satellite group of mice (n = 5) was euthanized and assessed for the extent of metastasis to the lungs by examining the number of surface lung macrometastases. PG545 significantly reduced the large metastases by 63% (>3 mm) but given the variability and small sample size did not significantly inhibit the number of small or medium sized metastases despite a reduction of 80% for medium- and 50% for small-sized metastases (Figure 3A). Histological assessment of micrometastases in the main group of animals revealed PG545 significantly reduced the percentage of sections with metastatic involvement in lung tissue (Figure 3B). Despite a single administration of PG545, four days prior to mastectomy, significantly inhibiting the growth of primary tumor (Figure 3C), no correlation between the size of individual tumors and the extent of micrometastases was found (Figure 3D) in the main group of mice (R^2^ = 0.1). Although not significant, the mean lung weight of the PG545-treated mice was approximately 36% lower than that of vehicle control-treated mice and similar to that of non-inoculated, untreated mice (data not shown). A correlation between lung weight and total metastases count for the satellite animals was demonstrated (R^2^ = 0.8042; Figure S3).

### PG545 Significantly Enhances Survival in the 4T1 Mastectomy Model in the Absence of an Inhibitory Effect on the Primary Tumor

**Figure 4:**
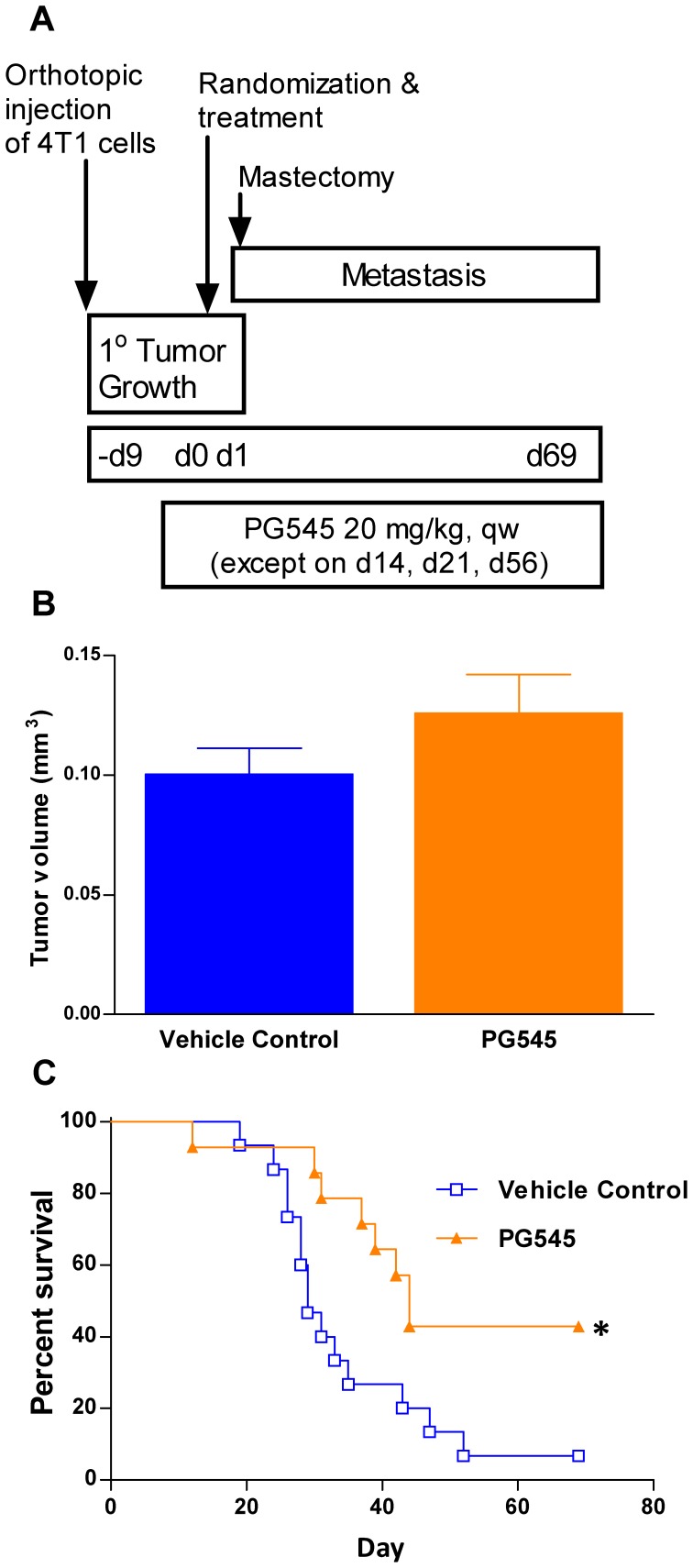
PG545 significantly enhances overall survival in the absence of effects on primary tumor size in 4T1 mastectomy model. (**A**) Experimental protocol for the mastectomy model in this instance delayed treatment with PG545 until one day prior to surgery to minimise a possible effect on primary tumor size. (**B**) PG545 administered one day prior to mastectomy had no inhibitory effect on the growth of the primary tumor. (**C**) The number of mice surviving at the end of the study was significantly greater in the PG545-treated group compared to vehicle control. The once weekly dosing regimen with PG545 resulted in an average bodyweight loss of 2.6% on day 68. Survival data were first analysed by Kaplan‐Meier analysis using Log ranks of survival. * = P<0.05 versus vehicle control.

Although the differences in size of the primary tumor at the time of mastectomy observed in the 4T1 survival model is not correlative with the resultant number of lung metastasis (Figure 3D) or overall survival in this model (data not presented), a follow-up survival study revealed PG545 significantly enhanced overall survival despite having no effect on primary tumor growth due to withholding treatment until the day before mastectomy. In this experiment, treatment with PG545 commenced one day prior to mastectomy and continued at 20 mg/kg at weekly intervals except on certain dosing holidays (Figure 4A). PG545, administered the day prior to mastectomy, had no effect on primary tumor growth (Figure 4B). Nevertheless, treatment with PG545 significantly enhanced overall survival with 40% of animals surviving beyond 60 days (Figure 4C). At the end of study on day 68, treatment with PG545 showed a net bodyweight loss of 2.6%.

### PG545 Reduces the Expression of Heparanase in Primary Tumor and Metastatic Lung Tissue

**Figure 5:**
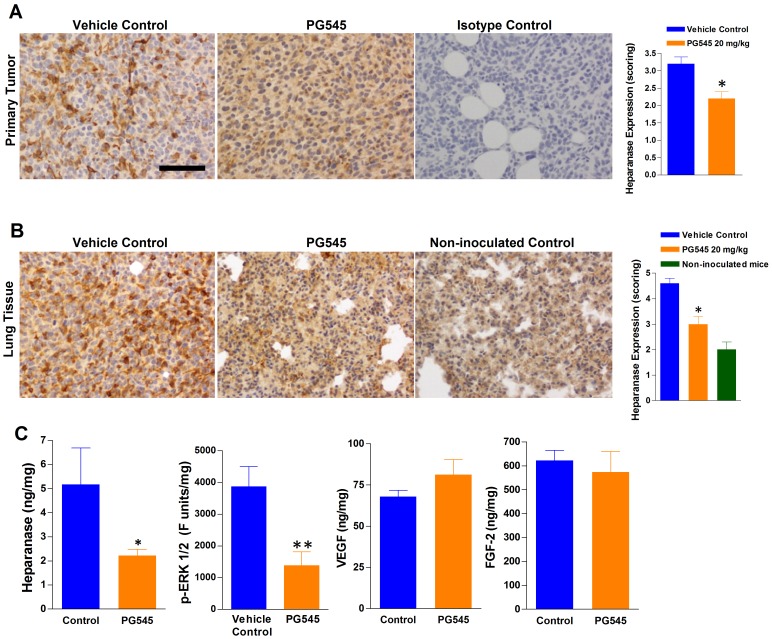
PG545 reduces the expression of heparanase in primary tumors and lung tissue. IHC images illustrate the reduction in the intensity of heparanase staining in primary tumor (**A**) which was surgically resected following a single administration of PG545 four days prior to mastectomy and in lung tissue (**B**) following multiple doses of PG545. Scale bar represents 50 µm for all images. The heparanase staining in the lungs of naive non-inoculated mice are also shown (**B** right panel). The histograms in (**A**) and (**B**) plot a semi-quantitative assessment of the IHC and they reveal that the heparanase staining was significantly reduced (* = P<0.05 versus relevant controls) in both the primary tumor and lung samples following treatment with PG545. (**C**) ELISA assays were used to quantify the levels of heparanase, phosphorylated ERK1/2, VEGF and FGF-2 in tumor tissue homogenates. The data are expressed as a function of total extracted protein. The results demonstrate that PG545 significantly reduced the levels of heparanase (P<0.05) and phosphorylated ERK1/2 (P<0.01) but not VEGF or FGF-2. Statistical comparisons to the vehicle control performed using a t-test.

Immunohistochemical staining for heparanase was detected in all 4T1 breast tumor and lung tissue, as well as in lung tissue from non-tumor bearing mice. Representative photographic images of heparanase stained sections of the primary tumor and lung for each group are presented in Figure 5A. The semi-quantitative score (conducted independently by two researchers in a blinded fashion) for heparanase staining was between 36–43% higher in diseased lung tissue compared with tumor tissue in 4T1-inoculated animals (Figure 5B). In tumor and lung tissue, heparanase expression was approximately 30% lower in the PG545-treated animals compared with the vehicle-treated animals. The heparanase staining in the sections was widespread associated with the ECM – in primary tumor tissue the majority of stained cells were tumor cells with some foamy macrophage staining whereas in the lung tissue, it was uniform throughout the ECM with minimal cytoplasmic staining and very few heparanase-positive macrophages. Heparanase was detected in the lung tissue from non-inoculated age-matched control mice, but comparing these levels with those in the tumor-bearing vehicle-treated mice indicates that heparanase was elevated more than double the basal level in the tumor-bearing mice.

The high expression of heparanase in the tumor tissue was confirmed using an ELISA method. Again, PG545 significantly reduced heparanase expression in tumor tissue (Figure 5C) but the method failed to detect substantial heparanase levels in lung tissue (data not shown). Preliminary Western blotting data supported the findings that PG545 reduces heparanase protein expression in 4T1 breast primary tumor homogenates. (Figure S4). Analysis of the phosphorylation levels of ERK1/2 in tumor tissue indicated that there was significantly less p-ERK1/2 in PG545 treated tumours (Figure 5C). In contrast, no significant changes in the levels of VEGF and FGF-2 in tumors following PG545 treatment could be detected when measured by ELISA (Figure 5C).

## Discussion

Progress in developing treatments for metastatic disease remains slow and the developmental approaches being taken are currently similar to those used for primary tumors [39]. A significant challenge is to demonstrate that a treatment provides clinical benefit in the metastatic setting and this issue has been recently highlighted by the use of bevacizumab in metastatic breast cancer [1]. While debate continues regarding appropriate endpoints in clinical trials for first-line metastatic breast cancer [40], an angiogenesis inhibitor bevacizumab was associated with objective responses in about 10% of patients with metastatic breast cancer who had received treatment with conventional therapies. However, while significant improvements in response rates and prolongations in time to progression were observed in two Phase III trials, no survival differences were observed [41]. Other angiogenesis inhibitors such as sunitinib, which has also demonstrated single agent activity of about a 10% response rate in breast cancer, together with other tyrosine kinase inhibitors, namely sorafenib and pazopanib, are currently being tested in clinical trials for metastatic breast cancer in combination with a variety of agents [41]. From a preclinical perspective, the relatively recent findings that some anti-angiogenic agents have the capacity to accelerate metastasis under certain circumstances [24], [25] may help to explain why antiangiogenic agents have had a relatively minor impact on patient survival [42]. Moreover, growth delay of tumors at the subcutaneous site is not predictive for drug response of residual metastatic disease [43]. Given the impact of heparanase on tumor angiogenesis and metastasis [44] and the anti-metastatic activity of HS mimetics/heparanase inhibitors [20], [23], [25], [27], [28], [45], [46], PG545 was investigated in the 4T1 mastectomy model using sorafenib as an antiangiogenic comparator agent.

PG545, administered twice weekly via the subcutaneous route or sorafenib, administered daily via the oral route, significantly inhibited the growth of the primary tumor. Once the primary tumor reached an average volume of 1500 mm^3^, the study was terminated and mice were assessed for the number of surface lung metastases. PG545 dose-dependently reduced the number of lung metastases while in contrast, sorafenib significantly increased the number of lung metastases. This latter finding with sorafenib is not unprecedented given previous reports of similar effects using other murine models of metastasis [28], [34], [35]. In the mastectomy model, PG545 (administered once weekly) but not sorafenib (administered daily), significantly enhanced overall survival. More detailed analyses on the PG545 treatment groups from the survival study confirmed a significant effect on primary tumor size at mastectomy and a lower number of macrometastases and micrometastases compared with the vehicle control group. Despite evidence showing that primary tumor weight does not necessarily correlate with number of metastases in the 4T1 model or in other breast cancer models [47], another study which administered PG545 one day rather than four days prior to mastectomy (thus not impacting the size of the primary tumor) revealed that PG545 continued to exert a significant effect on overall survival in this model. PG545 has been shown to be efficacious against subcutaneous tumours [27], [28], reduced lung colonisation in a B16 model [27] and also reduced spontaneous metastasis, albeit not using an orthotopic model [28], but this is the first study to show that it is active against a spontaneously-metastasizing orthotopic tumor model. Moreover, the 4T1 model has particular clinical relevance, firstly, due to its propensity to metastasize to sites affected in human breast cancer and, secondly, because longer studies employing survival as an endpoint can be performed using this model.

The data shown herein supports the hypothesis that in addition to experimental conditions, differential efficacies with antiangiogenic therapy may be observed between micro and macrometastatic disease and such distinctions could be important particularly in the adjuvant setting [48]. Given there are currently over forty adjuvant trials underway involving multiple antiangiogenic agents (including sorafenib), these aforementioned distinctions may become apparent in the clinic. Some preclinical models in which primary tumors are removed prior to treatment with angiogenesis inhibitors have indicated that metastatic growth can be inhibited or accelerated, depending on the tumor models, the drugs used, and when the treatments are initiated [48]. These different outcomes by anti-cancer agents led to a proposal by experts in the field that all antiangiogenic and other compounds in preclinical development be tested for efficacy in at least one metastasis model – preferably incorporating metastasis from an orthotopic site [42].

The current study demonstrated a potentially intriguing difference between two classes of antiangiogenic agent, namely the HS mimetic PG545 and the TKI sorafenib, in terms of primary tumor growth, anti-metastatic activity and overall survival following resection of the primary tumor. Interestingly, *in vitro* studies found no significant differences between PG545 and sorafenib in terms of inhibition of invasiveness (IC_50_) using 4T1 cells or human umbilical vein endothelial cells (Figure S5). A key difference between these classes of compounds, however, is PG545’s ability to inhibit the enzymatic function of heparanase *in vitro* [27]. Selective inhibition of heparanase by a HS mimetic was found to attenuate metastasis in a melanoma model expressing high levels of this endoglycosidase, but had no effect on the metastasis of a carcinoma cell line that expresses little or no heparanase activity [23]. Here, we examined the effect of PG545 on heparanase protein expression using samples from primary tumor tissue (taken at mastectomy) and lung tissue during the survival studies and found that PG545 significantly inhibited the expression of heparanase in both tumor tissue and lung tissue. The data indicate that in addition to its activity against the enzymatic activity of heparanase, PG545 also acts on heparanase by suppressing its expression, probably by inhibiting the signalling of one or both of VEGF and FGF-2. Inhibition of these growth factors would reduce activation of the ERK1/2 pathway which is consistent with the observed reduction in levels of phosphorylated ERK1/2 in the tumor tissue of PG545 treated mice. Both of these growth factors have been shown to stimulate heparanase expression [29], [30] and their inhibition in this experiment by PG545 appears to have driven down heparanase levels in the primary tumors and in the lungs where metastases are concentrated. Studies of tumor cells and T lymphocytes have shown that heparanase expression can be induced by the transcription factor early growth response 1 (EGR1) which, itself, is dependent on ERK1/2 activity [49], [50]. If heparanase expression in this model was under the same control, PG545 inhibition of growth factor-stimulated ERK activation would have lead to heparanase down-regulation as observed. Such down regulation of heparanase expression raises two important points about the anti-cancer properties of PG545: firstly, it will complement the inhibition of the enzyme’s activity by PG545 and, secondly, it will also reduce the signalling of heparanase [12], [13], something that other enzymatic inhibitors may not accomplish. These data are the first to provide direct evidence that PG545 inhibits tumor signalling *in vivo* and these observations are consistent with the previously proposed mechanism of action of this HS mimetic.

In contrast to heparanase, the absolute levels of VEGF and FGF-2 in the tumors did not appear to be significantly altered after four days of PG545 treatment which would, if our hypothesis based on p-ERK1/2 levels is correct, suggest that inhibition of VEGF and FGF-2 by PG545 does not suppress their own expression. This observation indicates that there is specificity in the mechanism whereby PG545 suppresses heparanase expression. In addition, the unchanged levels of VEGF and FGF-2 in PG545-treated and control tumors strongly suggest that the observed reduction in p-ERK1/2 was not due to a reduction in total ERK1/2, rather it was due to reduced signalling through the ERK pathway, probably caused by PG545 inhibition of one or both of VEGF and FGF-2.

During the mastectomy 4T1 studies there was evidence that administration of either PG545 or sorafenib combined with surgery might lead to an additive effect on bodyweight loss. Non-significant weight loss was also seen at high doses of PG545 (25 mg/kg) in the non-surgical study (Figure 1B). A comparison of PG545 studies across mouse, rat and dog indicates that these effects seem to be isolated to mouse.

In conclusion, PG545 not only inhibits the growth of primary tumors but also possesses potent anti-metastatic activity which leads to significantly enhanced overall survival in a mastectomy model of breast cancer. These findings are timely, given recent concerns about the paucity of preclinical studies addressing such issues [31]. Moreover, PG545 down regulates the expression of heparanase in primary tumor tissue and metastatic lung tissue thereby supporting the notion that targeting this enzyme at least contributes to, if not is directly responsible for, the anti-metastatic properties associated with this agent. Subject to the satisfactory completion of toxicology studies using intravenous administration, it is intended that PG545 re-enters clinical trials using this route of administration after an earlier trial was halted due to unexpected injection site reactions when administered subcutaneously. As PG545 progresses in the clinic, due consideration should be made for its investigation in micrometastatic disease settings, especially given the negligible effects of overall survival reported to date in clinical trials with other antiangiogenic agents and limited therapeutic options to address metastatic involvement in indications such as breast cancer.

## Supporting Information

Figure S1 **Cisplatin (1.4 mg/kg qw IV) significantly enhances overall survival in 4T1 mastectomy model.** (a) experimental protocol for the mastectomy model. Female Balb/c mice were injected with 10 µL of 1×10^5^ 4T1 cells into the fourth mammary fat pad and were randomised (n = 20 per group), based on tumour size, five days post-inoculation (Day 0). Cisplatin was first administered on Day 0 and led to a body weight loss of 5% by the end of study. The number of mice surviving at the end of the study was not significantly different compared with the PG545-treated group.
(PDF)

Figure S2 **Repeated weekly doses of PG545 are tolerated and reach acceptable exposure levels in mice.** PG545 was administered at 20 mg/kg (qwx3) or 20 mg/kg followed by 10 mg/kg (qwx2) to compare the bodyweight profiles (left panel). Plasma concentrations were collected at the estimated Tmax of 4 hours in mice (right panel) to check whether a dose reduction would impact the previously referenced efficacious exposure level (based solely on Cmax) of 20 µg/mL (Dredge et al 2011). Samples from the mice dosed at 20 mg/kg are not shown (due to operator error on day 8).
(PDF)

Figure S3 **Correlation of lung weight versus metastases.** Total metastases counts for ten inoculated satellite mice in vehicle control or PG545-treated groups on day 30.
(PDF)

Figure S4 **Analysis of heparanase expression in representative tumor lysates.** Detection of the 50 kD form of recombinant heparanase was accomplished using the anti-HPA1 antibody in tumor tissue lysates (lanes 1–6). A higher intensity stained band, indicating an increased amount of heparanase protein, was observed in the tumor lysates from the vehicle treated mice (PBS) (lanes 1–2), compared with PG545 treated animals (lanes 3–6).
(PDF)

Figure S5 **PG545 and sorafenib display similar effects in an in vitro invasion assay using 4T1 cells and HUVECs.** The human umbilical vein endothelial cell line HUVEC (pooled, EGM-2) were sourced from Lonza (Basel, Switzerland). The Glioblastoma cell line U87-MG was sourced from ATCC (Rockville, MD, USA) and was used to generate conditioned medium for use as the chemoattractant in the invasion assays. HUVEC were cultured in EGM-2 supplemented with the growth factors supplied in Lonza’s BulletKit. Conditioned medium was obtained from U87-MG cells (vP batch # 10012) cultured in MEM cell culture medium supplemented with 10% FBS, 100 IU/mL penicillin-streptomycin, 2 mM GlutaMax, and 0.1 mM NEAA. All cell lines were cultured at 37°C in a humidified cell culture incubator supplied with 95% air/5% CO_2_. For invasion assays, cell culture inserts (8 µm pore size) were washed twice in serum-free RPMI medium and placed into the wells of a 24-well cell culture plate. Using cold pipette tips, 40 µL of Matrigel diluted 1∶10 in RPMI medium were added to each insert. Matrigel was allowed to solidify by incubating overnight at 37°C. The following day, 4T1 cells or HUVECs were added to the upper chamber of each insert. Cell suspensions contained PG545 or Sorafenib at various concentrations and analysed in triplicate. U87-MG conditioned medium was added to the bottom chamber as the chemoattractant and the plates were incubated for 24 hours to allow the cells to migrate. The non-migrating cells were removed from the upper chamber and cells on the bottom side of the membrane were fixed and stained with 10% filtered Giemsa stain for 1 hour. Migration was quantified by counting cells in three fields of view at 40X magnification using an inverted light microscope (Olympus Australia, Mount Waverley, Victoria, Australia).The average number of migrated cells in three fields and three replicates were calculated. The percentage of inhibition of invasion was calculated using the formula: Inhibition (%) = [1− (Number of migrated cells at a given concentration/Number of migrated cells in untreated samples)] ×100% inhibition was plotted against concentration of PG545 or Sorafenib. A logarithmic scale was used for the axis containing the concentrations of PG545 or Sorafenib (x-axis).The 50% Inhibitory Concentration (IC_50_) was calculated using BioDataFit 1.02 (Chang Bioscience, Inc., Castro Valley, CA 94552, USA). A four parameter or sigmoidal approximation was used.
(PDF)

Table S1 **Toxicities associated with PG545 in mouse, rat and dog studies.**
(PDF)

## References

[1] Mina LA, Sledge GW (2011) Rethinking the metastatic cascade as a therapeutic target. Nat Rev Clin Oncol 8: 325–332. 21502993 10.1038/nrclinonc.2011.59

[2] Siegel R, Ward E, Brawley O, Jemal A (2011) Cancer statistics, 2011: The impact of eliminating socioeconomic and racial disparities on premature cancer deaths. CA Cancer J Clin 61: 212–236. 21685461 10.3322/caac.20121

[3] O’Shaughnessy J (2005) Extending survival with chemotherapy in metastatic breast cancer. Oncologist 10 (Suppl 3)20–29. 16368868 10.1634/theoncologist.10-90003-20

[4] Hayat MJ, Howlader N, Reichman ME, Edwards BK (2007) Cancer statistics, trends, and multiple primary cancer analyses from the Surveillance, Epidemiology, and End Results (SEER) Program. Oncologist 12: 20–37. 17227898 10.1634/theoncologist.12-1-20

[5] Jones SE (2008) Metastatic breast cancer: the treatment challenge. Clin Breast Cancer 8: 224–233. 18650152 10.3816/CBC.2008.n.025

[6] Verma S, McLeod D, Batist G, Robidoux A, Martins IRS, et al (2011) In the end what matters most? A review of clinical endpoints in advanced breast cancer. Oncologist 16: 25–35. 10.1634/theoncologist.2010-0278 PMC3228053 21212428

[7] Hammond E, Bytheway I, Ferro V (2006) Heparanase as a target for anticancer therapeutics: New developments and future prospects. In: Delehedde M, editor. New Developments in Therapeutic Glycomics. Trivandrum: Research Signpost. 251–282.

[8] McKenzie E (2007) Heparanase: a target for drug discovery in cancer and inflammation. Br J Pharmacol 151: 1–14. 17339837 10.1038/sj.bjp.0707182 PMC2012981

[9] Barash U, Cohen-Kaplan V, Dowek I, Sanderson RD, Ilan N, et al (2010) Proteoglycans in health and disease: new concepts for heparanase function in tumor progression and metastasis. FEBS J 277: 3890–3903. 20840586 10.1111/j.1742-4658.2010.07799.x PMC3000436

[10] Dempsey LA, Brunn GJ, Platt PJ (2000) Heparanase, a potential regulator of cell-matrix interactions. Trends Biochem Sci 25: 349–351. 10916150 10.1016/s0968-0004(00)01619-4

[11] Elkin M, Ilan N, Ishai-Michaeli R, Friedmann Y, Papo O, Pecker I (2001) Heparanase as mediator of angiogenesis: mode of action. FASEB J 15: 1661–1663. 11427519 10.1096/fj.00-0895fje

[12] Zetser A, Bashenko Y, Edovitsky E, Levy-Adam F, Vlodavsky I (2006) Heparanase induces vascular endothelial growth factor expression: correlation with p38 phosphorylation levels and Src activation. Cancer Res 66: 1455–1463. 16452201 10.1158/0008-5472.CAN-05-1811

[13] Ben-Zaken O, Gingis-Velitski S, Vlodavsky I (2007) Heparanase induces Akt phosphorylation via a lipid raft receptor. Biochem Biophys Res Commun 361: 829–834. 17689495 10.1016/j.bbrc.2007.06.188 PMC2390716

[14] Maxhimer JB, Quiros RM, Stewart R, Dowlatshahi K, Gattuso P, et al (2002) Heparanase-1 expression is associated with the metastatic potential of breast cancer. Surgery 132: 326–333. 12219030 10.1067/msy.2002.125719

[15] Imada T, Matsuoka J, Nobuhisa T, Okawa T, Murata T, et al (2006) COX-2 induction by heparanase in the progression of breast cancer. Int J Mol Med 17: 221–228. 16391819

[16] Götte M, Yip GW (2006) Heparanase, hyaluronan, and CD44 in cancers: a breast carcinoma perspective. Cancer Res 66: 10233–10237. 17079438 10.1158/0008-5472.CAN-06-1464

[17] Cohen I, Pappo O, Elkin M, San T, Bar-Shavit R, et al (2006) Heparanase promotes growth, angiogenesis and survival of primary breast tumors. Int J Cancer 118: 1609–1617. 16217746 10.1002/ijc.21552

[18] Maxhimer JB, Pesce CE, Stewart RA, Gattuso P, Prinz RA, Xu X (2005) Ductal carcinoma in situ of the breast and heparanase-1 expression: a molecular explanation for more aggressive subtypes. J Am Coll Surg 200: 328–335. 15737842 10.1016/j.jamcollsurg.2004.10.034

[19] Theodoro TR, Luongo de Matos L, Sant Anna AVL, Fonseca FLA, Semedo P, et al (2007) Heparanase Expression in Circulating Lymphocytes of Breast Cancer Patients Depends on the Presence of the Primary Tumor and/or Systemic Metastasis. Neoplasia 9: 504–510. 17603633 10.1593/neo.07241 PMC1899258

[20] Raman K, Kuberan B (2010) Chemical Tumor Biology of Heparan Sulfate Proteoglycans. Curr Chem Biol 4: 20–31. 20596243 10.2174/187231310790226206 PMC2892923

[21] Zhang L, Sullivan PS, Goodman JC, Gunaratne PH, Marchetti D (2011) MicroRNA-1258 suppresses breast cancer brain metastasis by targeting heparanase. Cancer Res 71: 645–654. 21266359 10.1158/0008-5472.CAN-10-1910 PMC3078691

[22] Liu C-J, Lee P-H, Lin D-Y, Wu C-C, Jeng L-B, et al (2009) Heparanase inhibitor PI-88 as adjuvant therapy for hepatocellular carcinoma after curative resection: a randomized phase II trial for safety and optimal dosage. J Hepatol 50: 958–968. 19303160 10.1016/j.jhep.2008.12.023

[23] Borsig L, Vlodavsky I, Ishai-michaeli R, Torri G, Vismara E (2011) Sulfated Hexasaccharides Attenuate Metastasis by Inhibition of P-selectin. Neoplasia 13: 445–452. 21532885 10.1593/neo.101734 PMC3084621

[24] Ritchie JP, Ramani VC, Ren Y, Naggi A, Torri G, et al (2011) SST0001, a chemically modified heparin, inhibits myeloma growth and angiogenesis via disruption of the heparanase/syndecan-1 axis. Clin Cancer Res 17: 1382–1393. 21257720 10.1158/1078-0432.CCR-10-2476 PMC3060291

[25] Zhou H, Roy S, Cochran E, Zouaoui R, Chu CL, et al (2011) M402, a novel heparan sulfate mimetic, targets multiple pathways implicated in tumor progression and metastasis. PloS One 6: e21106. 21698156 10.1371/journal.pone.0021106 PMC3116871

[26] De Bock K, Mazzone M, Carmeliet P (2011) Antiangiogenic therapy, hypoxia, and metastasis: risky liaisons, or not? Nat Rev Clin Oncol 8: 393–404. 21629216 10.1038/nrclinonc.2011.83

[27] Dredge K, Hammond E, Davis K, Li CP, Liu L, et al (2010) The PG500 series: novel heparan sulfate mimetics as potent angiogenesis and heparanase inhibitors for cancer therapy. Invest New Drugs 28: 276–283. 19357810 10.1007/s10637-009-9245-5

[28] Dredge K, Hammond E, Handley P, Gonda TJ, Smith MT, et al (2011) PG545, a dual heparanase and angiogenesis inhibitor, induces potent anti-tumour and anti-metastatic efficacy in preclinical models. Br J Cancer 104: 635–642. 21285983 10.1038/bjc.2011.11 PMC3049593

[29] Sasaki M, Ito T, Kashima M, Fukui S, Izumiyama N, et al (2001) Erythromycin and clarithromycin modulation of growth factor-induced expression of heparanase mRNA on human lung cancer cells in vitro. Mediators Inflamm 10: 259–267. 11759110 10.1080/09629350120093731 PMC1781717

[30] Luan Q, Sun J, Li C, Zhang G, Lv Y, et al (2011) Mutual enhancement between heparanase and vascular endothelial growth factor: a novel mechanism for melanoma progression. Cancer Lett 308: 100–111. 21624769 10.1016/j.canlet.2011.04.019

[31] Ebos JML, Kerbel RS (2011) Antiangiogenic therapy: impact on invasion, disease progression, and metastasis. Nat Rev Clin Oncol 8: 210–221. 21364524 10.1038/nrclinonc.2011.21 PMC4540336

[32] Holland SJ, Pan A, Franci C, Hu Y, Chang B, et al (2010) R428, a selective small molecule inhibitor of Axl kinase, blocks tumor spread and prolongs survival in models of metastatic breast cancer. Cancer Res 70: 1544–1554. 20145120 10.1158/0008-5472.CAN-09-2997

[33] Lelekakis M, Moseley JM, Martin TJ, Hards D, Williams E, et al (1999) A novel orthotopic model of breast cancer metastasis to bone. Clin Exp Metastasis 17: 163–170. 10411109 10.1023/a:1006689719505

[34] Ebos JML, Lee CR, Cruz-Munoz W, Bjarnason GA, Christensen JG, Kerbel RS (2009) Accelerated metastasis after short-term treatment with a potent inhibitor of tumor angiogenesis. Cancer Cell 15: 232–239. 19249681 10.1016/j.ccr.2009.01.021 PMC4540346

[35] Pàez-Ribes M, Allen E, Hudock J, Takeda T, Okuyama H, et al (2009) Antiangiogenic therapy elicits malignant progression of tumors to increased local invasion and distant metastasis. Cancer Cell 15: 220–231. 19249680 10.1016/j.ccr.2009.01.027 PMC2874829

[36] Korpal M, Ell BJ, Buffa FM, Ibrahim T, Blanco MA, et al (2011) Direct targeting of Sec23a by miR-200s influences cancer cell secretome and promotes metastatic colonization. Nat Med 17: 1101–1108. 21822286 10.1038/nm.2401 PMC3169707

[37] Utsugi T, Shibata J, Sugimoto Y (1996) Antitumor Activity of a Novel Podophyllotoxin Derivative (TOP-53) against Lung Cancer and Lung Metastatic Cancer Antitumor Activity of a Novel Podophyllotoxin Cancer and Lung Metastatic Cancer. Cancer Res 56: 2809–2814. 8665518

[38] Workman P, Aboagye EO, Balkwill F, Balmain A, Bruder G, et al (2010) Guidelines for the welfare and use of animals in cancer research. Br J Cancer 102: 1555–1577. 20502460 10.1038/sj.bjc.6605642 PMC2883160

[39] Sleeman J, Steeg PS (2010) Cancer metastasis as a therapeutic target. Eur J Cancer 46: 1177–1180. 20307970 10.1016/j.ejca.2010.02.039 PMC6545591

[40] Sargent D, Hayes D (2008) Assessing the measure of a new drug: is survival the only thing that matters? J Clin Oncol 26: 1922–1933. 18421044 10.1200/JCO.2007.14.8064

[41] Hortobagyi G, Esserman L, Buchholz T (2010) Neoplasms of the breast. In: Holland-Frei, editor. Cancer Medicine. Shelten, Connecticut: PMPH. 1449.

[42] Steeg PS, Anderson RL, Bar-Eli M, Chambers AF, Eccles SA, et al (2009) Preclinical Drug Development Must Consider the Impact on Metastasis. Clin Cancer Res 15: 4529–4530. 25278743 10.1158/1078-0432.CCR-09-1363 PMC4179907

[43] Day C-P, Carter J, Bonomi C, Hollingshead M, Merlino G (2012) Preclinical therapeutic response of residual metastatic disease is distinct from its primary tumor of origin. Int J Cancer 130: 190–199. 21312195 10.1002/ijc.25978 PMC3161145

[44] Vlodavsky I, Abboud-Jarrous G, Elkin M, Naggi A, Casu B, et al (2006) The impact of heparanese and heparin on cancer metastasis and angiogenesis. Pathophysiol Haemost Thromb 35: 116–127. 16855356 10.1159/000093553

[45] Li Q-N, Liu H-Y, Xin X-L, Pan Q-M, Wang L, et al (2009) Marine-derived oligosaccharide sulfate (JG3) suppresses heparanase-driven cell adhesion events in heparanase over-expressing CHO-K1 cells. Acta Pharmacol Sin 30: 1033–1038. 19543297 10.1038/aps.2009.97 PMC4006649

[46] Gandhi N, Mancera R (2010) Heparin/heparan sulphate-based drugs. Drug Discov Today 15: 1058–1069. 20974281 10.1016/j.drudis.2010.10.009

[47] Le Dévédec SE, van Roosmalen W, Maria N, Grimbergen M, Pont C, et al (2009) An improved model to study tumor cell autonomous metastasis programs using MTLn3 cells and the Rag2(−/−) gammac (−/−) mouse. Clin Exp Metastasis 26: 673–684. 19466569 10.1007/s10585-009-9267-6 PMC2776159

[48] Ebos JML, Lee CR, Kerbel RS (2009) Tumor and host-mediated pathways of resistance and disease progression in response to antiangiogenic therapy. Clin Cancer Res 15: 5020–5025. 19671869 10.1158/1078-0432.CCR-09-0095 PMC2743513

[49] deMestre A, Khachigian L, Santiago F, Staykova M, Hulett M (2003) Regulation of Inducible Heparanase Gene Transcription in Activated T Cells by Early Growth Response 1. J Biol Chem 278: 50677–50685. 10.1074/jbc.M310154200 14522979

[50] de Mestre AM, Rao S, Hornby JR, Soe-Htwe T, Khachigian LM, Hulett MD (2005) Early growth response gene 1 (EGR1) regulates heparanase gene transcription in tumor cells. J Biol Chem 280: 35136–35147. 16093249 10.1074/jbc.M503414200

