# Supplementary figures and images for: PG545, a Heparan Sulfate Mimetic, Reduces Heparanase Expression In Vivo, Blocks Spontaneous Metastases and Enhances Overall Survival in the 4T1 Breast Carcinoma Model
Source: PLoS One. 2012 Dec 26;7(12):e52175. doi: 10.1371/journal.pone.0052175 (PMC3530599; doi:10.1371/journal.pone.0052175)

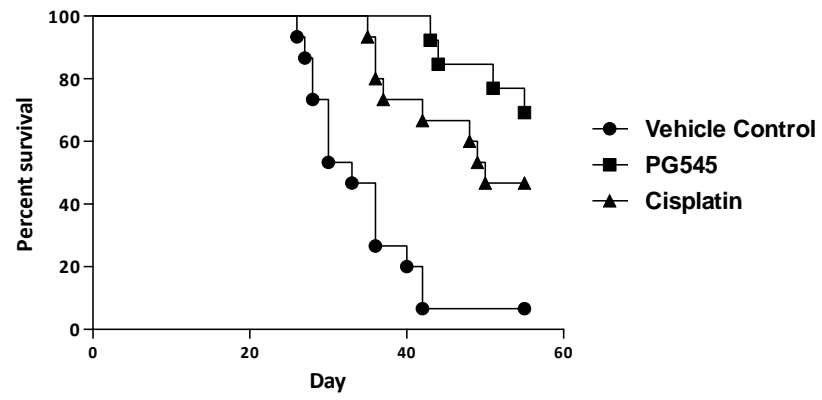

Supplement: Figure S1 — Cisplatin (1.4 mg/kg qw IV) significantly enhances overall survival in 4T1 mastectomy model. (a) experimental protocol for the mastectomy model. Female Balb/c mice were injected with 10 µL of 1×10^5^ 4T1 cells into the fourth mammary fat pad and were randomised (n = 20 per group), based on tumour size, five days post-inoculation (Day 0). Cisplatin was first administered on Day 0 and led to a body weight loss of 5% by the end of study. The number of mice surviving at the end of the study was not significantly different compared with the PG545-treated group. (PDF) [file pone.0052175.s001.pdf]

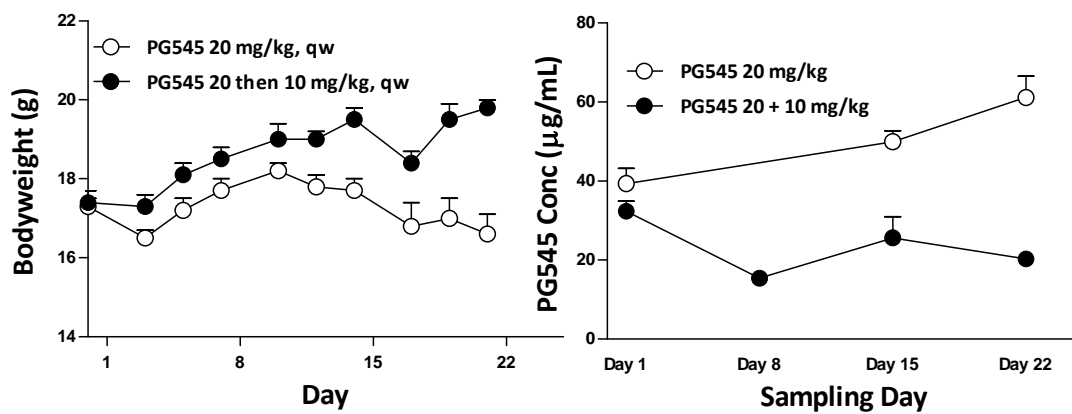

Supplement: Figure S2 — Repeated weekly doses of PG545 are tolerated and reach acceptable exposure levels in mice. PG545 was administered at 20 mg/kg (qwx3) or 20 mg/kg followed by 10 mg/kg (qwx2) to compare the bodyweight profiles (left panel). Plasma concentrations were collected at the estimated Tmax of 4 hours in mice (right panel) to check whether a dose reduction would impact the previously referenced efficacious exposure level (based solely on Cmax) of 20 µg/mL (Dredge et al 2011). Samples from the mice dosed at 20 mg/kg are not shown (due to operator error on day 8). (PDF) [file pone.0052175.s002.pdf]

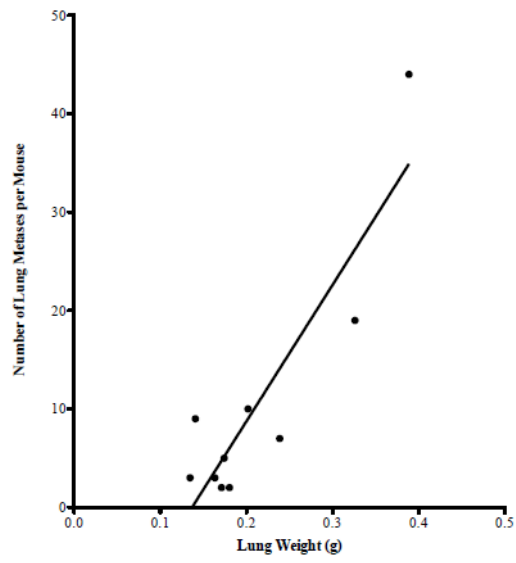

$R^2 = 0.8042$

Supplement: Figure S3 — Correlation of lung weight versus metastases. Total metastases counts for ten inoculated satellite mice in vehicle control or PG545-treated groups on day 30. (PDF) [file pone.0052175.s003.pdf]

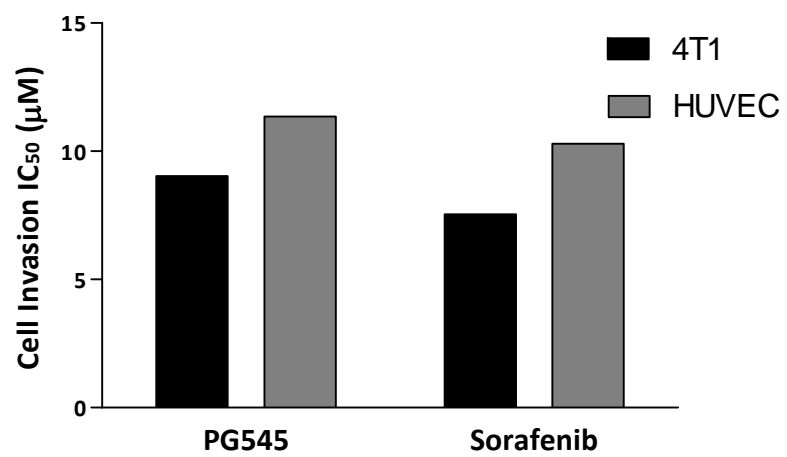

Supplement: Figure S5 — PG545 and sorafenib display similar effects in an in vitro invasion assay using 4T1 cells and HUVECs. The human umbilical vein endothelial cell line HUVEC (pooled, EGM-2) were sourced from Lonza (Basel, Switzerland). The Glioblastoma cell line U87-MG was sourced from ATCC (Rockville, MD, USA) and was used to generate conditioned medium for use as the chemoattractant in the invasion assays. HUVEC were cultured in EGM-2 supplemented with the growth factors supplied in Lonza’s BulletKit. Conditioned medium was obtained from U87-MG cells (vP batch # 10012) cultured in MEM cell culture medium supplemented with 10% FBS, 100 IU/mL penicillin-streptomycin, 2 mM GlutaMax, and 0.1 mM NEAA. All cell lines were cultured at 37°C in a humidified cell culture incubator supplied with 95% air/5% CO2. For invasion assays, cell culture inserts (8 µm pore size) were washed twice in serum-free RPMI medium and placed into the wells of a 24-well cell culture plate. Using cold pipette tips, 40 µL of Matrigel diluted 1∶10 in RPMI medium were added to each insert. Matrigel was allowed to solidify by incubating overnight at 37°C. The following day, 4T1 cells or HUVECs were added to the upper chamber of each insert. Cell suspensions contained PG545 or Sorafenib at various concentrations and analysed in triplicate. U87-MG conditioned medium was added to the bottom chamber as the chemoattractant and the plates were incubated for 24 hours to allow the cells to migrate. The non-migrating cells were removed from the upper chamber and cells on the bottom side of the membrane were fixed and stained with 10% filtered Giemsa stain for 1 hour. Migration was quantified by counting cells in three fields of view at 40X magnification using an inverted light microscope (Olympus Australia, Mount Waverley, Victoria, Australia).The average number of migrated cells in three fields and three replicates were calculated. The percentage of inhibition of invasion was calculated using the formula: Inhibition (%) = [1− [file pone.0052175.s005.pdf]
