# Supplementary material for: PG545, a Heparan Sulfate Mimetic, Reduces Heparanase Expression In Vivo, Blocks Spontaneous Metastases and Enhances Overall Survival in the 4T1 Breast Carcinoma Model
Source: PLoS One. 2012 Dec 26;7(12):e52175. doi: 10.1371/journal.pone.0052175 (PMC3530599; doi:10.1371/journal.pone.0052175)
Supplement: Figure S4 — Analysis of heparanase expression in representative tumor lysates. Detection of the 50 kD form of recombinant heparanase was accomplished using the anti-HPA1 antibody in tumor tissue lysates (lanes 1–6). A higher intensity stained band, indicating an increased amount of heparanase protein, was observed in the tumor lysates from the vehicle treated mice (PBS) (lanes 1–2), compared with PG545 treated animals (lanes 3–6). (PDF) [file pone.0052175.s004.pdf]

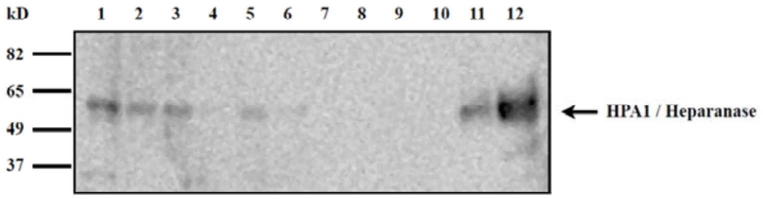

Lane	Description
1	ID: 37128 (Control, 20µg)
2	ID: 37128 (Control, 10µg)
3	ID: 37404 (PG545, 20µg)
4	ID: 37404 (PG545, 10µg)
5	ID: 76046 (PG545, 20µg)
6	ID: 76046 (PG545, 10µg)
7	Blank
8	HPA1 protein (0.2ng)
9	HPA1 protein (0.5ng)
10	HPA1 protein (1.0ng)
11	HPA1 protein (5.0ng)
12	HPA1 protein (10.0ng)
