# Supplementary material for: PG545, a Heparan Sulfate Mimetic, Reduces Heparanase Expression In Vivo, Blocks Spontaneous Metastases and Enhances Overall Survival in the 4T1 Breast Carcinoma Model
Source: PLoS One. 2012 Dec 26;7(12):e52175. doi: 10.1371/journal.pone.0052175 (PMC3530599; doi:10.1371/journal.pone.0052175)
Supplement: Table S1 — Toxicities associated with PG545 in mouse, rat and dog studies. (PDF) [file pone.0052175.s006.pdf]

Supplementary Table S1: Toxicities associated with PG545 in mouse, rat and dog studies.

Species	Study	Dose (mg/kg)	Frequency	Duration (weeks)	HED* (mg)	Bodyweight Change Over Study Duration (%)	Comments
Mouse	Tolerance/PK (naive)	20	q.w.	4	98	-4.5%	Piloerection and cold touch on days 2-3.
		20 (d1) & 10 (d8,16,22)	q.w.	4	98 (d1), &49 (d8, 16 & 22)	14.1	Piloerection and cold touch on days 2-3.
		15	q.w.	4	73	10.8	Piloerection and cold touch on days 2-3.
Mouse	4T1 study (non-surgical)	15	2 q.w.	2	73	5.2	Mild Piloerection.
		20	2 q.w.	2	98	-1.3	Mild Piloerection.
		25	2 q.w.	2	122	-4.7	Mild Piloerection.
Mouse	4T1 study (mastectomy/survival)	20 x2, 10 x6	q.w.	8	98 x2, 49 x 6	-11.8	Moderate Piloerection. Mild rapid breathing (day 33-55).
Mouse	Non-GLP Acute Toxicity	50	Single	2	826	2.5	No observable adverse effect level (NOAEL).
Rat	GLP Repeat Dose Toxicity	3	q.w.	4	29	26.4	NOAEL.
		12	q.w.	4	116	25.1	↑ cholesterol, triglycerides, WBC, liver & kidney weights associated with vacuolation of tissues, injection site reactions.
		48	q.w.	4	465	24.6	↑ cholesterol, triglycerides, WBC, APTT, liver & kidney weights associated with vacuolation of tissues, injection site reactions. ↓platelets.
Dog	GLP Repeat Dose Toxicity	2.5	q.w.	4	83	15.3	Lowest observable adverse effects level (LOAEL).
		7.5	q.w.	4	250	13.1	↑ temperature, cholesterol, triglycerides, liver & kidney weights associated with vacuolation of tissues, injection site reactions.
		20	q.w.	4	660	12.6	↑ temperature, cholesterol, triglycerides, WBC, APTT, liver & kidney weights associated with vacuolation of tissues, injection site reactions. ↓RBC indices and platelets.

* HED= Human Equivalent Dose taken from FDA Guidance for Industry on Estimating the Maximum Safe Starting Dose in Initial Clinical Trials for Therapeutics in Adult Healthy Volunteers (http://www.fda.gov/downloads/Drugs/.../Guidances/UCM078932.pdf)
